# Morphological characteristics of the infrapatellar fat pad

**DOI:** 10.1038/s41598-022-12859-1

**Published:** 2022-05-27

**Authors:** Mutsuaki Edama, Tomofumi Otsuki, Hirotake Yokota, Ryo Hirabayashi, Chie Sekine, Sae Maruyama, Ikuo Kageyama

**Affiliations:** 1 Institute for Human Movement and Medical Sciences, Niigata University of Health and Welfare, Shimami-cho 1398, Kita-ku, Niigata, 950-3198 Japan; 2 Department of Anatomy, School of Life Dentistry at Niigata, Nippon Dental University, Niigata, Japan

**Keywords:** Anatomy, Medical research

## Abstract

The relationship between the morphological characteristics of the infrapatellar fat pad (IFP) and joint deformity has yet to be fully elucidated. Therefore, the purpose of this study was to clarify the morphological characteristics of the IFP and to identify the relationships between morphological characteristics of the IFP and degenerative grade of the articular surface of the patella. This investigation examined 41 legs from 25 Japanese cadavers. The IFP length, width, and volume were measured. It was categorized into three types: Type I, IFP proximal located on medial and lateral sides of the patella; Type II, the IFP proximal only located medially; and Type III, absence of the IFP proximal. Articular surfaces were graded as macroscopically intact or mildly altered (Grade I), moderately (Grade II), or severely (Grade III). Grade III was significantly more frequent than Grades I or II in Type III. IFP volume was significantly larger in Type I than in Types II or III. A negative correlation was found between the degenerative grade of the articular surface of the patella and IFP volume. It was suggested that a relationship between the degenerative grade of the articular surface of the patella and the IFP volume.

## Introduction

The infrapatellar fat pad (IFP), also known as Hoffa’s fat pad^1^, fills the space in the knee joint, providing lubrication for the joint and ensuring that the space is maintained^2^. The IFP is an intracapsular, extrasynovial structure^3–5^. Histological investigation of the IFP has led to the suggestion that this structure acts as a pressure absorber during knee articulation and that the relatively high density of nerves within the IFP may indicate a mechanoreceptor/proprioceptor role for the IFP^6^. Furthermore, together with the plica of the knee, the IFP has been suggested to provide internal support for the patella, mirroring the support given by the external retinacula^7^. The IFP contains free nerve terminals and pain transmitters, and so is considered one cause of knee joint pain^8^. In recently, the new findings suggest that inflammation of IFP and synovial membrane within the knee may have a central role in OA pain and may drive peripheral and central sensitization in Knee OA^9,10^.

The morphological characteristics of the IFP, in terms of exact size, shape and volume, appear highly variable. Previous anatomical studies have been performed on a small number of cadavers^1,3,5,11^, using magnetic resonance imaging^12–17^, ultrasonography^18^, and arthroscopy^19^. Focusing on gross anatomical research, Gallagher et al.^5^ reported that the IFP attaches to the infrapatellar pole, patellar tendon, meniscus, and anterior tibia. In recent years, the existence of the IFP proximal located on the medial and lateral sides of the patella has been reported. In a report examining 36 knees from fresh cadavers^20^, IFP superior medial extension was present in 100%, IFP superior lateral extension in 83%, and a loop-type IFP in 11%. In a report using 43 knees from formalin-fixed bodies^3^, IFP superior medial extension was present in 81%, IFP superior lateral extension in 65%, and loop type in 9.3%. Mean IFP volume has been reported as 24 ml (range 12–36 ml)^5^, 26.86 ± 6.82 ml^11^, and 29.2 ± 6.1 ml^20^. Thus, the morphological characteristics of the IFP have yet to be fully investigated.

In recent years, the IFP has been suggested to play an important role in the initiation and progression of knee osteoarthritis (OA)^21,22^. Biochemically, the IFP can produce various pro-inflammatory cytokines and adipokines, which may be deleterious to the knee joint^23,24^. Pathological examination of IFP tissue obtained from patients with severe OA revealed the presence of vascular neoformations, fibrosis, and chronic inflammation in these specimens^25^. In another study, individuals with abnormal signal intensity in the IFP and/or greater volume of effusion synovitis in the absence of radiographic OA knee were suggested to be at greater risk of accelerated OA knee, which may be characterized by local inflammation^26^. And, Cowan et al.,^27^ reported that individuals with patellofemoral joint osteoarthritis (PFJ OA) had a larger IFP than non-OA controls, and IFP volume was directly related to PFJ OA pain. In contrast, other data have suggested that a larger IFP maximum area is protective against cartilage defects and pain, potentially through the IFP contributing to a shock absorbing mechanism in the knee^28,29^. Therefore, the relationship between the morphological characteristics of IFP and joint deformity has yet to be fully elucidated.

The purpose of this study was to clarify the morphological characteristics of IFP (shape, length, width, volume) using Japanese cadavers and to identify the relationships between morphological characteristics of the IFP (shape, volume) and degenerative grade of the articular surface of the patella.

## Results

### Classification of the IFP

Type I was seen in 15 knees (37%), Type II in 20 knees (49%), and Type III in 6 knees (14%). Loop type was present in 1 knee (3%) in Type I. In determining differences between left and right legs, we were able to measure both legs from 16 cadavers (32 legs). No significant differences were apparent between males and females or between left and right sides (Table 1).

**Table 1:** Comparison of IFP classifications by sex and laterality.

	Type I	Type II	Type III
Male (n = 18)	6 (15)	9 (22)	3 (7)
Female (n = 23)	9 (22)	11 (27)	3 (7)
Total	15 (37)	20 (49)	6 (14)
Right (n = 16)	4 (13)	10 (31)	1 (3)
Left (n = 16)	7 (21)	6 (19)	4 (13)
Total	11 (34)	16 (50)	5 (16)

Values are reported as number of specimens (%).

### Morphological characteristics of the IFP

IFP superior medial extension length was significantly longer than IFP superior lateral extension length in Type I (p = 0.002). IFP superior medial extension distal width was significantly longer than IFP superior lateral extension distal width in Type I (p < 0.001). IFP volume was significantly higher in Type I than in Type II (p = 0.013) or Type III (p = 0.028) (Table 2).

**Table 2:** Morphological characteristics of the IFP.

	IFP superior medial extension (mm)				IFP superior lateral extension (mm)				IFP body (mm)		IFP volume (ml)
	Length	Width			Length	Width			Length	Width	
		Distal	Intermediate	Proximal		Distal	Intermediate	Proximal			
Type I	33 ± 12^a^	17 ± 6^b^	11 ± 3	23 ± 6	15 ± 11	6 ± 2	12 ± 4	25 ± 6	46 ± 9	83 ± 12	24 ± 5^c,d^
Type II	36 ± 10	18 ± 7	10 ± 2	23 ± 6					50 ± 7	75 ± 9	19 ± 4
Type III									42 ± 6	76 ± 7	18 ± 3

Values represent mean ± standard deviation.

^a^p = 0.002 vs. length of IFP superior lateral extension.

^b^p < 0.001 vs. distal width of IFP superior lateral extension.

^c^p = 0.013 vs. IFP volume of Type II.

^d^p = 0.028 vs. IFP volume of Type III.

### Relationship between degenerative grade of the articular surface of the patella and the classification of the IFP

In Type I, Grade I was seen in 9 knees (22%), Grade II in 1 knee (3%), and Grade III in 5 knees (12%). In Type II, Grade I was seen in 7 knees (17%), Grade II in 10 knees (24%), and Grade III in 3 knees (7%). In Type III, Grade I was seen in 1 knee (3%), Grade II in 0 knees (0%), and Grade III in 5 knees (12%). There was significant difference degenerative grade of the articular surface and the classification of the IFP (p = 0.003). In Type I, residual analysis indicated there were significantly lower in Grade II than in Grades I and III (adjusted residual − 2.2). In Type II, residual analysis indicated there were significantly higher in Grade II than in Grades I and III (adjusted residual 3.3). In Type III, residual analysis indicated there were significantly higher in Grade III than in Grades I and II (adjusted residual 2.9) (Table 3).

**Table 3:** Relationship between degenerative grade of articular surface of patella and IFP.

	Type I	Type II	Type III
Grade I	9 (22)	7 (17)	1 (3)
Grade II	1 (3)^a^	10 (24)^b^	0 (0)
Grade III	5 (12)	3 (7)	5 (12) ^c^
Total	15 (37)	20 (48)	6 (15)

Values represent number of specimens (%).

^a^p = 0.003 vs. grade I and grade III.

^b^p = 0.003 vs. grade I and grade III.

^c^p = 0.003 vs. grade I and grade II.

### The correlation of between degenerative grade of the articular surface of the patella and IFP volume

**Figure 1:**
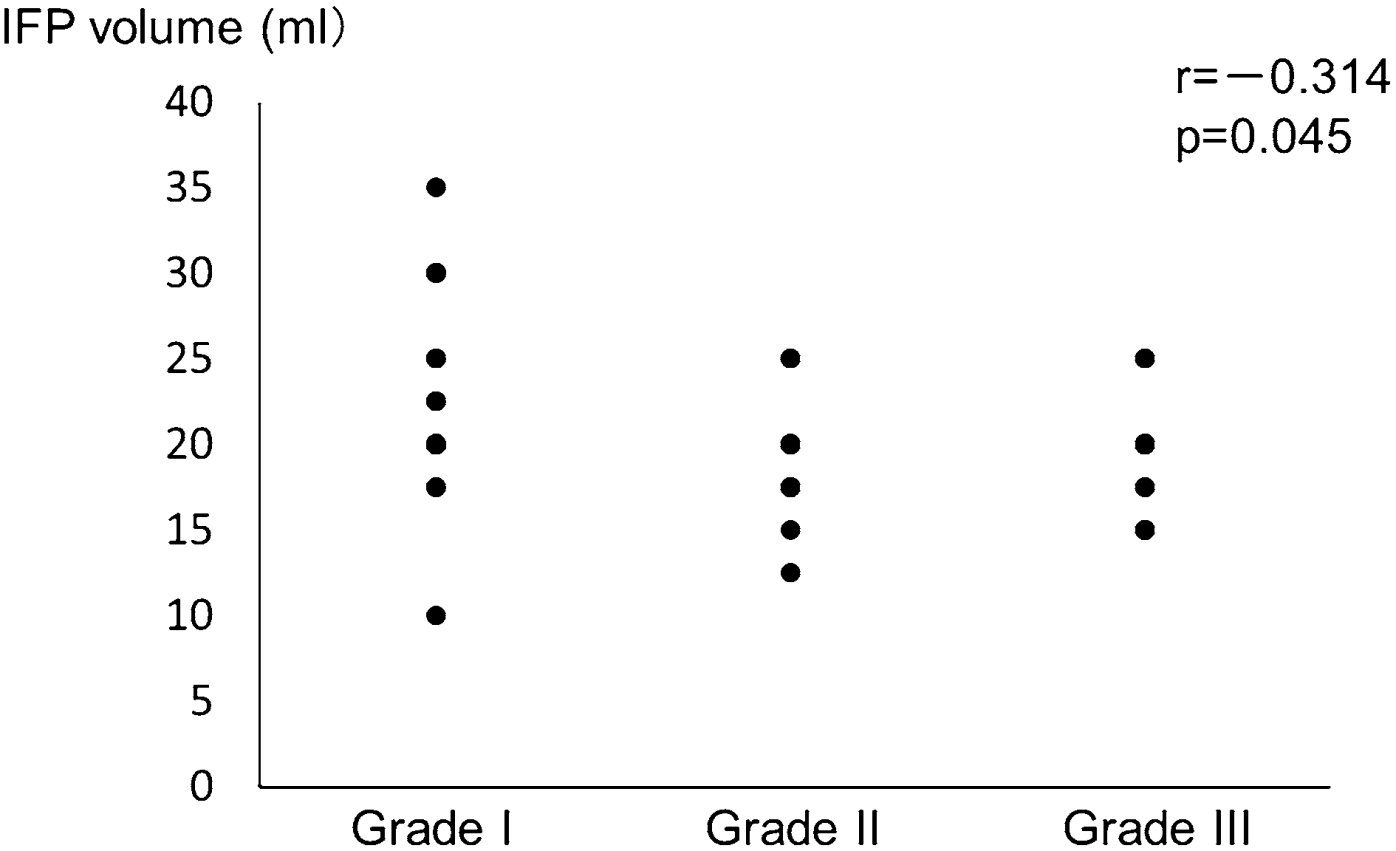
The correlation of between degenerative grade of the articular surface of the patella and IFP volume. Grade I: macroscopically intact or only mildly altered; Grade II: macroscopically moderately altered (if fissuring or fibrillation is observed); Grade III: macroscopically severely altered (if eburnation is present).

Negative correlation was found between degenerative grade of the articular surface of the patella and IFP volume (r = − 0.314, p = 0.045) (Fig. 1).

## Discussion

This study intended to clarify the morphological characteristics of IFP (shape, length, width, volume) using Japanese cadavers and the relationship between morphological characteristics of IFP (shape, volume) and degenerative grade of the articular surface of the patella. To the best of our knowledge, no detailed anatomical studies of the relationship between morphological characteristics of the IFP and degeneration of the articular surface of the patella have been reported previously.

In this study, Type I was seen in 15 knees (37%), Type II in 20 knees (49%), and Type III in 6 knees (14%). The IFP superior medial extension was seen in 35 knees (86%), IFP superior lateral extension in 15 knees (37%), and loop type in 1 knee (3%). In previous studies, the existence of the IFP proximal located on the medial and lateral sides of the patella has been reported^3,20^. In a study using 36 knees from fresh cadavers^20^, IFP superior medial extension was seen in 100%, IFP superior lateral extension in 83%, and loop type in 11%. In a study using 43 knees from formalin-fixed bodies^3^, IFP superior medial extension was seen in 81%, IFP superior lateral extension in 65%, and loop type in 9.3%. The result was that the frequencies of IFP superior lateral extension and loop type were low. The reason for this might be due to differences in specimens (ethnic background, cadaver fixation method) and methods of dissection.

In this study, Grade III was significantly more frequent than Grades I or II in Type III. And IFP volume was significantly larger in Type I than in Types II or III. In addition, weak negative correlation was found between degenerative grade of the articular surface of the patella and IFP volume. In previous studies, Cowan et al.,^27^ reported that individuals with PFJ OA had a larger IFP than non-OA controls, and IFP volume was directly related to PFJ OA pain. In contrast, Fontanella et al.,^17^ reported that a progressive decrease of IFP depth, femoral and tibial length and an increase of hypointense signal were observed in MRI of moderate and end-stage OA compared to non-osteoarthritic controls suggesting an important role of inflammation derived fibrosis and dimensions in OA pathology. And other data have suggested that a protective role of the IFP in knee OA, with a larger IFP being associated with reduced development and progression of knee pain and cartilage volume loss over^28,29^. In addition, Steidle-klok et al.,^30^ reported that Independent of the ambiguous role of the IFP in knee osteoarthritis, the size of the IFP does not appear to be related to knee pain. Interestingly, different findings on the relationship between IFP volume and degeneration of the PFJ have been reported, although the subjects, analysis methods, and study designs differ. In this study, it was suggested that a relationship to the degenerative grade of the articular surface of the patella and the IFP volume. This appears a quite interesting observation that must be deepen in the future.

Some limitations should be considered when interpreting the findings from this investigation. First, there are evaluation items. This study doesn’t evaluate the femoral trochlea surface. In addition, degeneration of the articular surface of the patella often be accompanied by osteophytes^31^, which would seem to affect the proximal IFP. But this study doesn’t evaluate osteophyte formation. And patella baja has been reported to cause patellar ligament shortening, fibrosis of the Hoffa’s fat pad, and other complications^31^. But this study doesn’t evaluate the patellar alignment. In the future, there is a need to investigate to correlate the IFP volume with both degeneration of joint surface (the patellar surface and the femoral trochlea surface) and osteophyte formation in the patellofemoral joint. Furthermore, an in vivo prospective study using ultrasound or MRI examination is needed including patellar alignment. Second, this study was to clarify the morphological characteristics of IFP in Japanese cadavers, but bilateral and unilateral knees are mixed. This study does not adequately reflect the Japanese knees. In addition, as all cadavers were from Japanese individuals, whether the present findings apply to individual from other ethnicities is unclear. Further studies are required to evaluate variations according to ethnic origin.

## Methods

### Cadavers

This investigation examined 41 legs from 25 Japanese cadavers (mean age at death, 80 ± 12 years; range 47–96 years; 18 sides from men, 23 from women; 20 right sides, 21 left sides) that had been switched to alcohol after placement in 10% formalin. No knees showed any signs of previous major surgery around the knee in any specimens. This study was approved by the ethics committee at our institution (approval number: 18430).

### Measurement conditions

**Figure 2:**
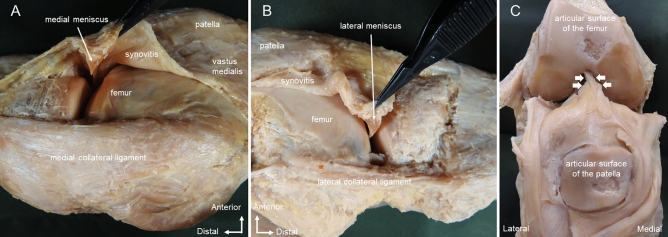
Method of dissection; right knee. (**A**) Medial view. The synovium and medial meniscus are incised along the anterior edge of the medial collateral ligament, and the synovium and meniscus are lifted forward. (**B**) Lateral view. The synovium and lateral meniscus are incised along the anterior edge of the lateral collateral ligament, and the synovium and meniscus are lifted forward. (**C**) Anterior view. Knee flexion position. The quadriceps femoris and patella are inverted from proximal to distal. White arrow: ligamentum mucosum.

**Figure 3:**
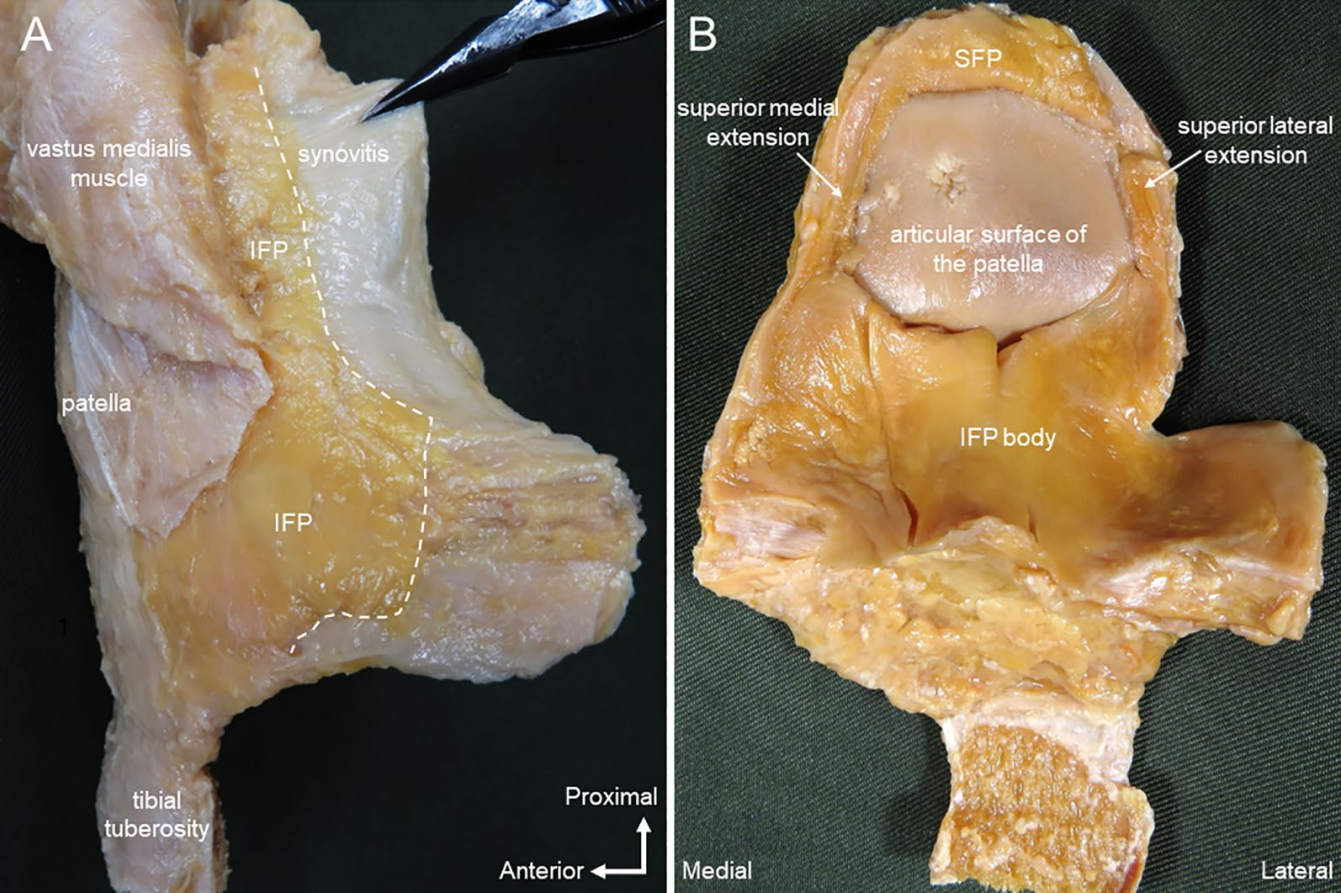
Method for dissection of the infrapatellar fat pad; right knee. (**A**) Medial view. The vastus medialis muscle is flipped forward and the synovium is gripped with forceps. An incision is made along the white dashed line to remove synovium not adherent to the infrapatellar fat pad. (**B**) Posterior view. The quadriceps femoris is removed along the morphology of the infrapatellar fat. *IFP* infrapatellar fat pad, *SFP* suprapatellar fat pad. White dashed line: boundary between Infrapatellar fat pad and synovitis.

**Figure 4:**
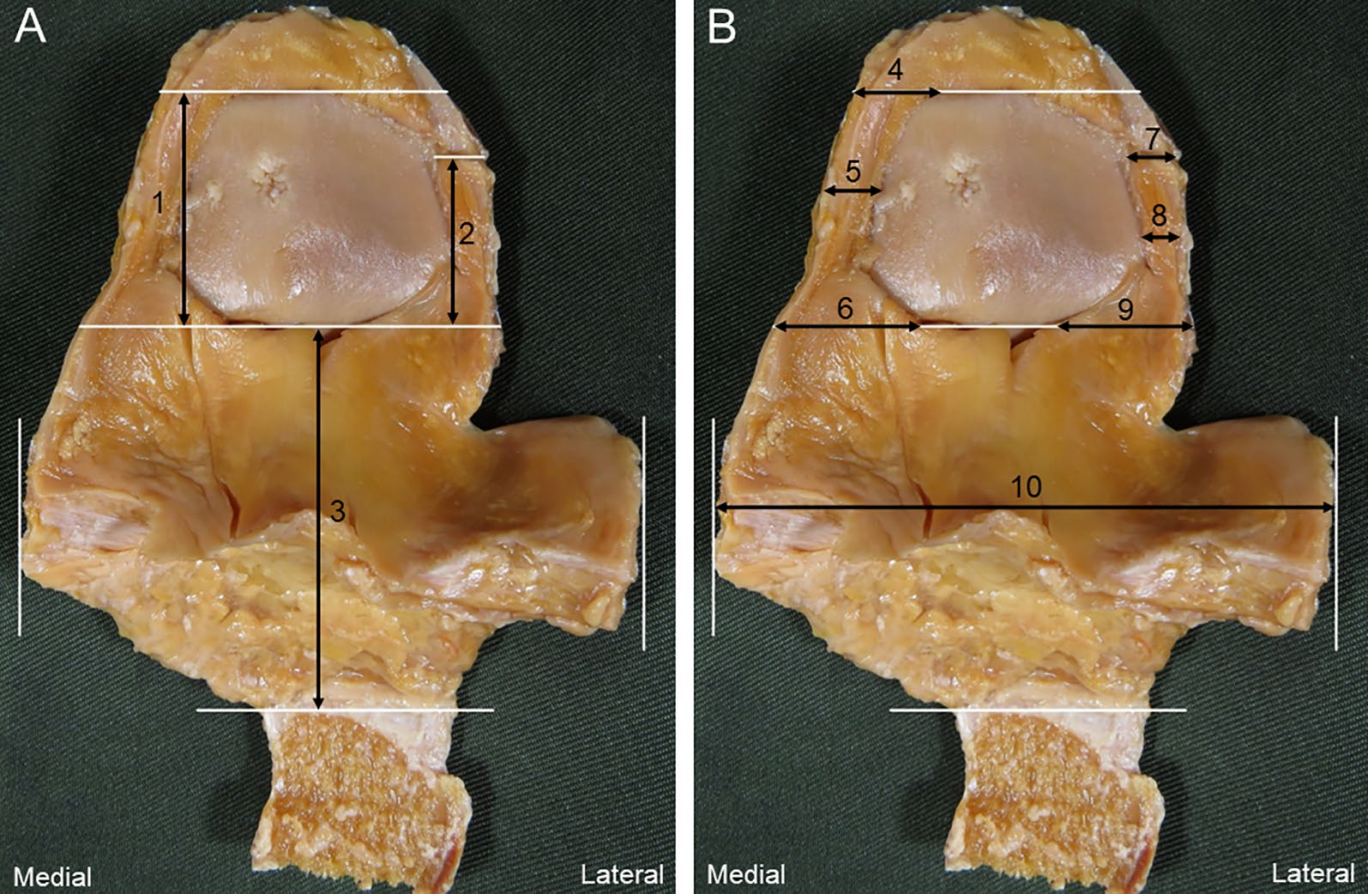
Measurement of the infrapatellar fat pad; right knee, posterior view. (**A**) Length of the infrapatellar fat pad superior medial extension and superior lateral extension, infrapatellar fat pad body. (**B**) Width of the infrapatellar fat pad superior medial extension and superior lateral extension, infrapatellar fat pad body. Black arrow 1: In cases with the infrapatellar fat pad superior medial extension connecting to the suprapatellar fat pad. Black arrow 2: In cases with the infrapatellar fat pad superior lateral extension not connecting to the suprapatellar fat pad. Black arrow 3: Infrapatellar fat pad body. Black arrow 4: Infrapatellar fat superior medial extension top. Black arrow 5: Infrapatellar fat superior medial extension center. Black arrow 6: Infrapatellar fat superior medial extension end. Black arrow 7: Infrapatellar fat superior lateral extension top. Black arrow 8: Infrapatellar fat superior lateral extension center. Black arrow 9: Infrapatellar fat superior lateral extension end. Black arrow 10: Infrapatellar fat pad body. White line: Reference line.

In the dissection procedure, an isolated knee specimen was prepared by cutting the distal one-third of the femur and the central part of the tibia, and the skin, subcutaneous tissue, and tensor fasciae lateral were removed. For the IFP body, the synovium and meniscus were incised along the anterior edges of the medial collateral ligament (MCL) and lateral collateral ligament (LCL) (Fig. 2A,B). Then, in a position of knee flexion, the quadriceps femoris and patella were inverted from proximally to distally, and the ligamentum mucosum was incised (Fig. 2C). The IFP was detached from the anterior surface of the tibia, and the patellar tendon was resected with the patellar tendon attached to the patella and part of the rough surface of the tibia. The meniscus and transverse knee ligament attached to the IFP were then carefully removed. The presence or absence of the IFP proximal was confirmed between the insertional tendons of the vastus medialis/vastus lateralis, the patellar retinaculum, and the synovium (Fig. 3). Regarding the removal of the synovium, only synovium that was not adherent to the IFP proximal and IFP body was carefully removed (Fig. 3A). Quadriceps muscle fiber bundles were removed along the shape of the IFP proximal (Fig. 3B). Part of the tibial tuberosity was carefully removed from the posterior part to remove the most distal end of the IFP body. After measuring the IFP superior medial extension and superior lateral extension and IFP body, the IFP was removed from the patella, patellar tendon, and patellar retinaculum for IFP volume measurement. With reference to previous studies^11^, these values weren't corrected by body size. Morphological measurement was performed by one examiner, and the IFP superior medial extension and superior lateral extension, IFP body length, width, and IFP volume were measured (Fig. 4). The specimen was photographed from behind using a digital camera (Finepix F600EXR; Fujifilm, Tokyo, Japan) with the patella joint surface facing forward without tilting. At the time of measurement, perpendicular lines were drawn in the following parts: the upper end of the patellar articular surface; the most end of the IFP superior medial extension and superior lateral extension, the lower end of the patellar articular surface, the horizon passing through the distal end of the IFP body, and the medial and lateral ends of the IFP body. IFP superior medial extension and superior lateral extension length was measured as the distance between the horizontal line passing through the upper end of the patellar joint surface and the horizontal line passing through the lower end of the patellar joint surface when the IFP was connected to the suprapatellar fat pad (SFP). When the IFP did not bind to the SFP, this distance was taken as the distance between the horizontal line passing through the most end of the IFP proximal and the horizontal line passing through the lower end of the patellar articular surface (Fig. 4A). IFP superior medial extension and superior lateral extension width was measured at the distance of the horizontal line connecting the medial and lateral ends of the IFP superior medial extension and superior lateral extension length and was measured at three points of the IFP superior medial extension and superior lateral extension length (Fig. 4B). IFP body length was measured as the distance between the horizontal line passing through the lower end of the patellar joint surface and the horizontal line passing through the most distal end of the IFP body. The IFP body width is the distance of the line connecting the perpendicular lines passing through the inner and outer ends of the IFP body. Image analysis software (Image J; NIH, Bethesda, MD, USA) was used for the measurement, and the mean ± standard deviation from three measurements was calculated. Before measuring volume, the IFP was soaked in a preservation solution (water: 78%, alcohol: 16%, glycerin: 4%, phenol: 2%) for 10 min to prevent the IFP from drying out. For the IFP volume, a 500-ml cylinder (SANPLATEC, Osaka, Japan) was used, and water displacement was recorded in 2.5-ml intervals, and the mean ± standard deviation from three measurements was calculated. When the IFP superior medial extension and SFP were connected, the SFP was removed at the upper end of the patellar joint surface. To prevent a decrease in IFP volume due to drying of the IFP, specimens were immersed in the preservation solution at 30-min intervals for 10 min, and the entire experiment was performed within 3 h.

**Figure 5:**
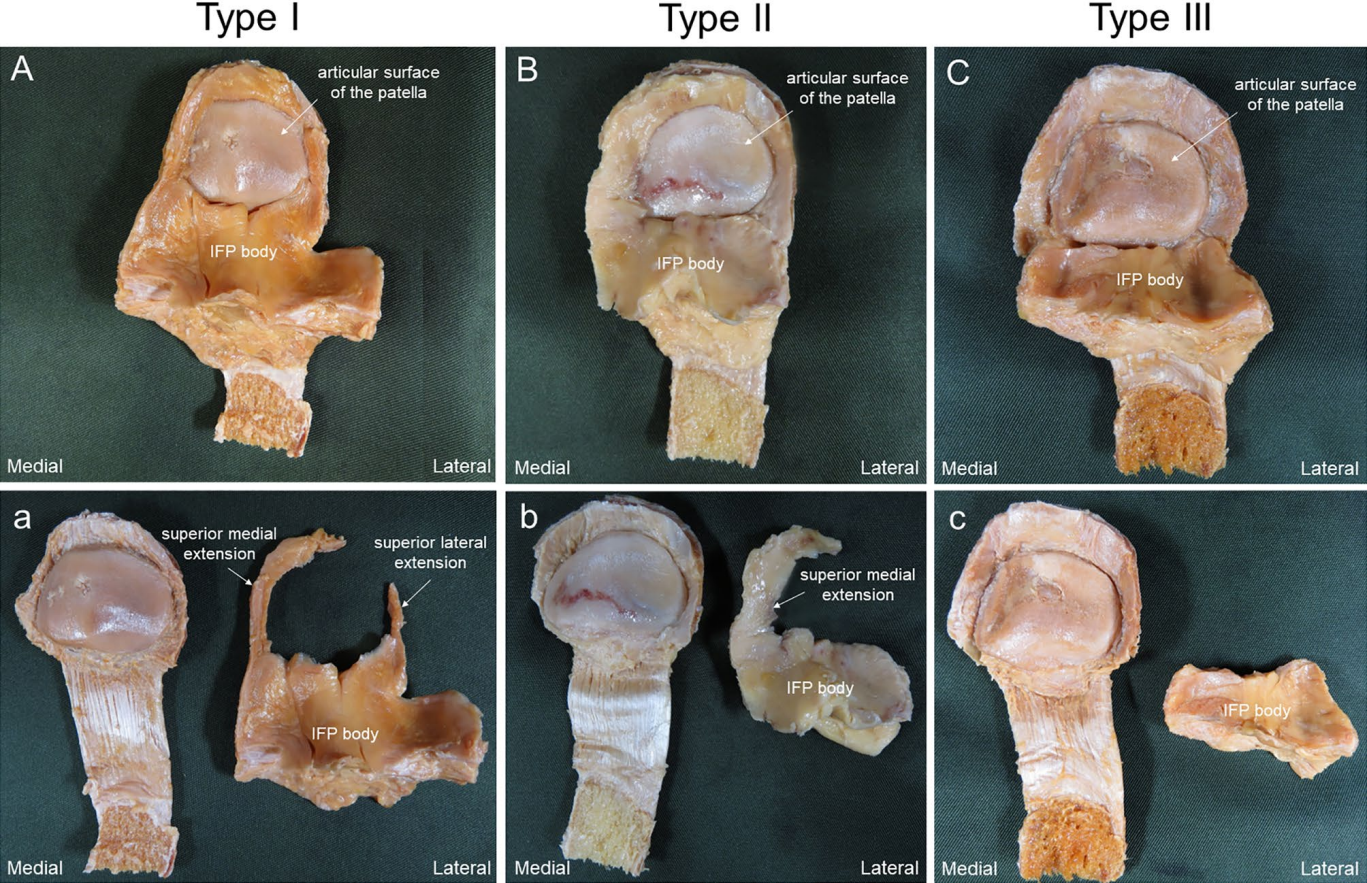
Classification of the IFP. (**A**) Type I: IFP proximal located on the medial side (superior medial extension) and lateral side (superior lateral extension) of the patella. (**B**) Type II: only IFP proximal located on the medial side (superior medial extension) of the patella. (**C**) Type III: absence of IFP proximal located on the medial and lateral sides of the patella. (**a**–**c**) IFP removed from the patella. *IFP* infrapatellar fat pad.

Knees were classified into 3 types by the morphological characteristics of IFP superior medial extension and superior lateral extension. Type I was an IFP located on the medial side (superior medial extension) and lateral side (superior lateral extension) of the patella (Fig. 5A,a). Type II was an IFP located only on the medial side (superior medial extension) of the patella (Fig. 5B,b). Type III was absence of the IFP on both superior medial extension and superior lateral extension (Fig. 5C,c).

**Figure 6:**
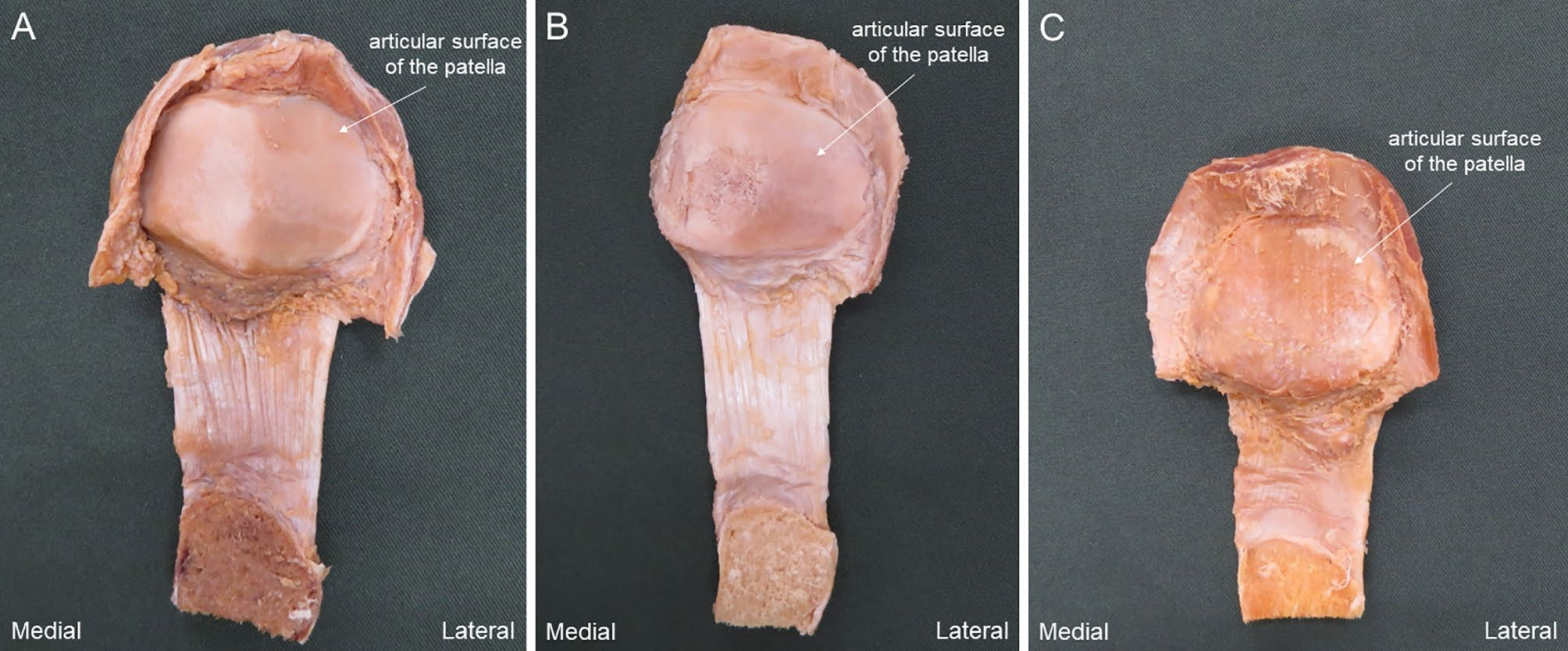
Classification of degenerative grade of the articular surface. (**A**) Grade I: macroscopically intact or only mildly altered. (**B**) Grade II: macroscopically moderately altered (if fissuring or fibrillation is observed). (**C**) Grade III: macroscopically severely altered (if eburnation is present).

The articular surfaces of the patella were graded^32^ as macroscopically intact or mildly altered (Grade I) (Fig. 6A), moderately altered (if fissuring or fibrillation was observed, Grade II) (Fig. 6B), or severely altered (if eburnation was present, Grade III) (Fig. 6C).

### Statistical analysis

The Shapiro–Wilk test was used to assess normality of the different variables. Fisher’s exact test was used for comparisons of classifications of the IFP by sex and laterality, and to compare differences in degenerative grade of the articular surface for each type of category. Residual analysis was used for the post hoc test. Comparisons of IFP superior medial extension length, width (distal, intermediate, proximal) between Types I and II were made using unpaired t-tests. Comparisons of IFP body length, width, and IFP volume in each type were made with one-way repeated-measures analysis of variance, and Tukey’s method. Comparisons of IFP superior medial extension and superior lateral extension length, width (distal, intermediate, proximal) among Type I were made with paired t-tests. Comparisons between degenerative grade of the articular surface of the patella and IFP volume were made using Spearman’s correlations. Statistical analyses were performed using SPSS version 26.0 software (SPSS Japan, Tokyo, Japan). The level of significance was p < 0.05.

### Ethics approval and consent to participate

All methods were carried out in accordance with the 1964 Declaration of Helsinki, and all cadavers were legally donated for research purposes to the Nippon Dental University, Niigata, Japan. Informed consent was obtained from the families of all subjects. This study was approved by the ethics committee of the Nippon University of Health and welfare (approval number: 18430, approval date: September 2019).

## Perspective

In this study, we clarified the morphological characteristics of the IFP using Japanese cadavers and the relationship between morphological characteristics of the IFP and degenerative grade of the articular surface of the patella. The IFP superior medial extension was seen in 35 knees (86%), IFP superior lateral extension in 15 knees (37%), and loop type in 1 knee (3%). It was suggested that a relationship between the degenerative grade of the articular surface of the patella and the IFP volume.

## Data Availability

The datasets generated and/or analysed during the current study are not publicly available due to limitations of ethical approval involving the patient data and anonymity but are available from the corresponding author on reasonable request.

## References

[1] Hoffa A (1904). The influence of the adipose tissue with regard to the pathology of the knee joint. J. Am. Med. Assoc..

[2] Mac CM (1950). The movements of bones and joints; The synovial fluid and its assistants. J. Bone Jt. Surg. Br..

[3] Duri ZA, Aichroth PM, Dowd G (1996). The fat pad. Clinical observations. Am. J. Knee Surg..

[4] Biedert RM, Sanchis-Alfonso V (2002). Sources of anterior knee pain. Clin. Sports Med..

[5] Gallagher J, Tierney P, Murray P, O'Brien M (2005). The infrapatellar fat pad: Anatomy and clinical correlations. Knee Surg. Sports Traumatol. Arthrosc..

[6] Macchi V (2016). The infrapatellar adipose body: A histotopographic study. Cells Tissues Organs.

[7] Geraghty RM, Spear M (2017). Evidence for plical support of the patella. J. Anat..

[8] Kohn D, Deiler S, Rudert M (1995). Arterial blood supply of the infrapatellar fat pad. Anatomy and clinical consequences. Arch. Orthop. Trauma Surg..

[9] Belluzzi E (2019). Contribution of infrapatellar fat pad and synovial membrane to knee osteoarthritis pain. Biomed. Res. Int..

[10] Inomata K (2019). Time course analyses of structural changes in the infrapatellar fat pad and synovial membrane during inflammation-induced persistent pain development in rat knee joint. BMC Musculoskelet. Disord..

[11] Leese J, Davies DC (2020). An investigation of the anatomy of the infrapatellar fat pad and its possible involvement in anterior pain syndrome: A cadaveric study. J. Anat..

[12] Saddik D, McNally EG, Richardson M (2004). MRI of Hoffa's fat pad. Skelet. Radiol..

[13] Ozkur A, Adaletli I, Sirikci A, Kervancioglu R, Bayram M (2005). Hoffa's recess in the infrapatellar fat pad of the knee on MR imaging. Surg. Radiol. Anat..

[14] Abreu MR, Chung CB, Trudell D, Resnick D (2008). Hoffa's fat pad injuries and their relationship with anterior cruciate ligament tears: New observations based on MR imaging in patients and MR imaging and anatomic correlation in cadavers. Skelet. Radiol..

[15] Ladenhauf HN (2020). Association of infra-patellar fat pad size with age and body weight in children and adolescents. Ann. Anat..

[16] Diepold J (2015). Sex-differences of the healthy infra-patellar (Hoffa) fat pad in relation to intermuscular and subcutaneous fat content-data from the Osteoarthritis Initiative. Ann. Anat..

[17] Fontanella CG (2019). Quantitative MRI analysis of infrapatellar and suprapatellar fat pads in normal controls, moderate and end-stage osteoarthritis. Ann. Anat..

[18] Naredo E (2021). Dynamic changes in the infrapatellar knee structures with quadriceps muscle contraction. An in vivo study. Ann Anat.

[19] Brooker B, Morris H, Brukner P, Mazen F, Bunn J (2009). The macroscopic arthroscopic anatomy of the infrapatellar fat pad. Arthroscopy.

[20] Stephen JM (2018). The infrapatellar fat pad is a dynamic and mobile structure, which deforms during knee motion, and has proximal extensions which wrap around the patella. Knee Surg. Sports Traumatol. Arthrosc..

[21] Clockaerts S (2010). The infrapatellar fat pad should be considered as an active osteoarthritic joint tissue: A narrative review. Osteoarthr. Cartil..

[22] Ioan-Facsinay A, Kloppenburg M (2013). An emerging player in knee osteoarthritis: The infrapatellar fat pad. Arthritis Res. Ther..

[23] Eymard F (2014). Induction of an inflammatory and prodegradative phenotype in autologous fibroblast-like synoviocytes by the infrapatellar fat pad from patients with knee osteoarthritis. Arthritis Rheumatol..

[24] Clockaerts S (2012). Cytokine production by infrapatellar fat pad can be stimulated by interleukin 1β and inhibited by peroxisome proliferator activated receptor α agonist. Ann. Rheum. Dis..

[25] Maculé F (2005). Hoffa's fat pad resection in total knee arthroplasty. Acta Orthop. Belg..

[26] Davis JE (2019). Effusion-synovitis and infrapatellar fat pad signal intensity alteration differentiate accelerated knee osteoarthritis. Rheumatology (Oxford).

[27] Cowan SM, Hart HF, Warden SJ, Crossley KM (2015). Infrapatellar fat pad volume is greater in individuals with patellofemoral joint osteoarthritis and associated with pain. Rheumatol. Int..

[28] Pan F (2015). A longitudinal study of the association between infrapatellar fat pad maximal area and changes in knee symptoms and structure in older adults. Ann. Rheum. Dis..

[29] Teichtahl AJ (2015). A large infrapatellar fat pad protects against knee pain and lateral tibial cartilage volume loss. Arthritis Res. Ther..

[30] Steidle-Kloc E (2018). Relationship between knee pain and infrapatellar fat pad morphology: A within- and between-person analysis from the osteoarthritis initiative. Arthritis Care Res. (Hoboken).

[31] Bei M (2019). A novel rat model of patellofemoral osteoarthritis due to Patella Baja, or low-lying Patella. Med. Sci. Monit..

[32] Noyes FR, Stabler CL (1989). A system for grading articular cartilage lesions at arthroscopy. Am. J. Sports Med..

